# NK cells from COVID-19 positive patients exhibit enhanced cytotoxic activity upon NKG2A and KIR2DL1 blockade

**DOI:** 10.3389/fimmu.2023.1022890

**Published:** 2023-07-07

**Authors:** Grace Lee, Robert Schauner, Juanita Burke, Jade Borocz, Smitha Vasana, Lukasz Sobieraj, Maria Giraudo, Zachary Jackson, Qasim Ansari, Maria Navas, Hany Sakr, David Wald

**Affiliations:** ^1^ Department of Pathology, Case Western Reserve University, Cleveland, OH, United States; ^2^ Department of Pathology, Louis Stokes Cleveland Veteran Affairs (VA) Medical Center, Cleveland, OH, United States; ^3^ Department of Pediatrics, University Hospitals Cleveland Medical Center, Cleveland, OH, United States; ^4^ Midwestern University Chicago College of Osteopathic Medicine, Downers Grove, IL, United States

**Keywords:** SARS CoV-2, COVID-19, NK cell immunophenotype, KIRs, NK

## Abstract

SARS CoV-2 has caused a global pandemic leading to significant morbidity and mortality. There is a need to elucidate and further understand the implications of COVID-19 disease on the immune system to develop improved therapeutic strategies. In particular, Natural Killer (NK) cells play an essential role in mediating the innate immune response against viral infections. To better understand the role of innate immunity in COVID-19, we characterized the phenotype of circulating NK cells from 74 COVID-19 patients and 25 controls. Through evaluating the protein expression of activating and inhibitory NK cell surface molecules using dimension reduction analysis and clustering, we identified 4 specific clusters of NK cells specific to disease state (COVID-19 positive or COVID-19 negative) and characterized COVID-19 positive NK cells as: NGK2A+KIR2DL1+NKG2C-. Utilizing blocking antibodies specific for receptors NKG2A and KIR2DL1, we found that both NKG2A and KIR2DL1 blockade markedly enhances the ability of NK cells from COVID-19 positive patients to lyse SARS-Cov-2 infected cells. Overall, this study reveals new insights into NK cell phenotypes during SARS-CoV-2 infection and suggests a therapeutic approach worthy of further investigation to enhance NK cell-mediated responses against the virus.

## Introduction

Severe Acute Respiratory Syndrome Coronavirus-2 (SARS CoV-2) is a pathogenic, highly transmissible novel beta coronavirus that is responsible for acute respiratory disease. The clinical manifestations of COVID-19 infection range from mild (low grade fever/headaches/myalgia/loss of smell and taste) to severe (pneumonia/acute respiratory distress syndrome (ARDS)/acute kidney injury/disseminated intravascular coagulation/multi organ dysfunction and death) (1). The clinical manifestations of COVID-19 are driven by an imbalanced immune response (2). For example, patients with severe disease exhibit elevated chemokine and cytokine levels, including an abnormal type 1 interferon response (3, 4). Further studies identify altered T and B cells in severe COVID-19, shedding light on the important aspects of immune response to COVID-19 (1, 5). However, our understanding of the disease is still lacking.

Natural killer (NK) cells, which are a part of the innate immune system, have long been linked to the immunological response against respiratory viral infections. They bridge the innate and adaptive immune system and execute effector functions promoting viral clearance. With the global surge in COVID-19 infections, the significance of NK cells in control of viral infection sparked interest (6). NK cells do not need prior antigen sensitization, hence fight viruses early in the innate immune response. This is accomplished by a variety of effector functions controlled by a dynamic balance of inhibitory and activating NK cell receptors (7). NK cells migrate to the lungs in response to increased cytokine expression [Tumor necrosis factor alpha (TNF-) and IL-6] from the COVID-19 infected pulmonary epithelial cells resulting in interferon gamma (IFN-γ) mediated lysis as well as antibody mediated cellular cytotoxicity (ADCC) *via* S-protein triggered killing of virus infected host cells (8).

There is now compelling evidence that lymphopenia is associated with severe COVID-19 manifestation. A large study analyzing the association of immunological features with COVID-19 severity revealed, significantly lower levels of circulating NK cells as well as B cells, and T cells in severe as compared to mild cases. Similar associations were observed with cytokines (TNF-,IL-5, IL-6, and IL-10) and chemokines [Monocyte Chemoattractant Protein-1 (MCP-1), IFN-γ, inducible protein-10 (IP-10) and eotaxin] (9, 10).

In addition to a decrease in NK cell numbers in patients with severe COVID-19 symptoms, some differences in NK cell phenotype have been reported in COVID-19 patients, suggesting that the NK cells are dysfunctional. For example, it has been reported that NK cells may be exhausted in COVID-19 patients due to overexpression of PD-1 and decreased expression of NKG2D (11). Other investigators have reported that NKG2A is elevated in more severe COVID-19 cases and that utilizing NKG2A blockade can enhance cytotoxicity of patient-derived NK cells in genetically modified spike protein-expressing cell models (12, 13). The expression of the molecules associated with NK cell activity including granzyme A, and have also been found to be increased in COVID-19 patients as compared to healthy controls (14, 15), while there are conflicting reports on changes in NKG2C expression (15, 16). While some previous studies have described abnormalities in NK cell count and phenotype in severe COVID-19, further studies are needed to develop strategies that may reverse this dysfunction in COVID-19 disease (17). Here we characterized NK cells derived from COVID-19 patients as well as controls to assess for differences in NK cell phenotype to reveal new strategies to enhance NK cell activity in patients infected with the virus.

## Results

### Study design, patient cohort, and lymphocyte composition in COVID-19

To profile the NK cell immunophenotype in COVID-19, we analyzed 100 peripheral blood samples collected during active disease from 100 subjects from the Louis Stokes Cleveland VA hospital (**Table 1**). All cases of COVID-19 were confirmed by PCR testing and disease severity was assigned based on the worst symptoms present at the time of blood draw, according to the National Institutes of Health COVID-19 Treatment Guidelines Panel. There were no significant differences in sex, race, ethnicity, age, or co-morbidities among the different COVID-19 severity groups as determined by chi-square analysis. However, consistent with the VA patient population, the samples were predominantly from white males between 60-89 years old. In addition to patients with active COVID-19, 25 COVID-19 negative patients as confirmed by PCR testing were used as a comparison cohort.

**Table 1:** Patient demographics and co-morbidities classified by disease status and severity.

		All	Covid-	Asymptomatic	Mild/Moderate	Severe/Critical	P-Value
		n= (100%)	n= (%)	n= (%)	n= (%)	n= (%)	
		99	25	13	45	16	
Sex	Male	93	24 (25.8)	13(14)	41 (44.1)	15 (16.1)	0.922
	Female	6	1 (16.7)	0 (0)	4 (66.7)	1 (16.7)	
Race	White	51	11 (21.6)	7 (13.7)	23 (45.1)	10 (19.6)	0.6
	Black/African American	38	10 (26.3)	4 (10.5)	20 (52.6)	4 (10.5)	
	Asian	1	0 (0)	0 (0)	1 (100)	0 (0)	
	Not reported	9	4 (44.4)	2 (22.2)	1 (11.1)	2 (22.2)	
Ethnicity	Non-hispanic	91	23 (25.3)	12 (13.2)	41 (45)	14 (15.4)	0.995
	Hispanic	4	0 (0)	1 (25)	2 (50)	1 (25)	
	Not reported	4	2 (8)	0 (0)	2 (4)	1 (6)	
Age Range	30-59	38	11 (28.9)	4 (10.5)	18 (47.4)	5 (13.1)	0.975
	60-89	58	14 (24.1)	9 (15.5)	25 (43.1)	10 (17.2)	
	90+	3	0 (0)	0 (0)	2 (66.7)	1 (33.3)	
Co-Morbidities	Chronic Lung Disease			1 (11.1)	7 (77.8)	1 (11.1)	0.8
	Cardiac Disease			9 (20)	25 (54.3)	12 (26)	
	Obesity			4 (14.3)	16 (57.1)	8 (28.6)	
	Diabetes			4 (17.4)	14 (60.9)	5 (21.7)	
	HIV			0 (0)	1 (100)	0 (0)	
	Cancer			4 (36.4)	5 (63.6)	2 (18.2)	

P-value calculated by chi-square test.

**Figure 1:**
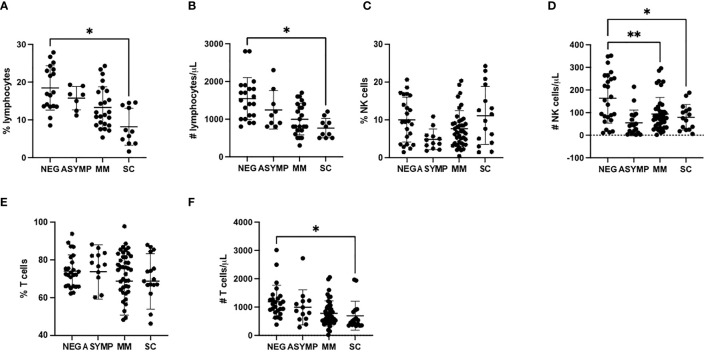
COVID-19 positive patients demonstrate reduced NK and T cells **(A, B)** Lymphocyte percentage **(A)** and number **(B)** decreases with increased disease severity. **(C)** NK cells percentage is unchanged with disease severity. **(D)** NK cell number decreases in COVID-19 positive patients. **(E)** T cells percentage is unchanged with increased disease severity. **(F)** T cell number decreases with increased disease severity. *P<0.05, **P<0.01.

To study the NK cell phenotype in patient samples, we analyzed peripheral blood mononuclear cells (PBMCs) using a 10-color NK cell receptor focused flow cytometry panel (**Table 2**). Due to shared clinical features, the severe (n=11) and critical (n=5) groups were combined in the analysis, as were the mild and moderate groups. Initially we assessed for changes in both the frequency and absolute number of lymphocytes among the cohorts. Consistent with previous observations from other studies, a decrease in the frequency and percentage of lymphocytes was observed with increased disease severity (**Figures 1A, B**). In particular, there was a notable decrease in absolute numbers of NK cells in comparing healthy controls to the Mild/Moderate (MM) and Severe/Critical (SC) groups (**Figures 1C, D**). Similarly, a significant decrease in the absolute number of T cells was also observed between healthy controls and the SC COVID-19 positive group (**Figures 1E, F**).

**Table 2:** Flow cytometry panel used for immunophenotyping.

Lasers	Color	Target	Alternate names	
FL1	FITC	CD159a	NKG2A	KLRC1
FL2	PE	CD335	Nkp46	NCR1
FL3	PE Vio615	CD314	NKG2D	KLRK1
FL4	PC5.5	CD158a_h	KIR2DL1/DS1	
FL5	PC7	CD158b1_b2	KIR2DL2/DL3	
FL6	APC	CD158e1_e2	KIR3DL1/DS1	
FL7	APC-A700	CD56		
FL8	APC-A750	CD16		
FL9	PB	CD94	NKG2c	KLRD1
FL10	KrO	CD3		

### Phenotypic assessment of NK cells in COVID-19

**Figure 2:**
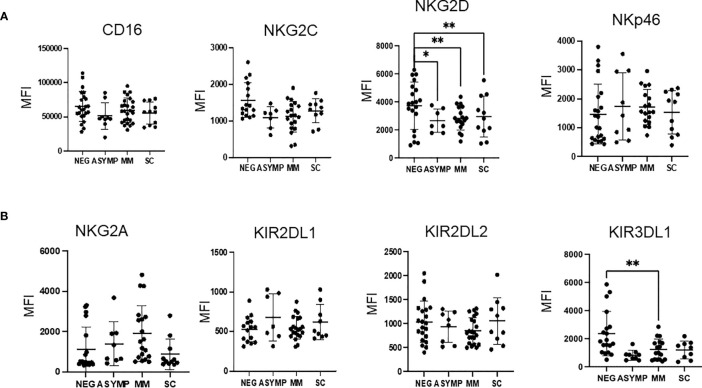
Changes in NK cell activating and inhibitory surface receptors observed by disease severity. **(A, B)** MFI of activating receptors **(A)** and inhibitory **(B)** receptors from peripheral blood NK cells. *P<0.05, **P<0.01.

In order to better appreciate differences in NK cells among the cohorts, we assessed the expression of a panel of activating and inhibitory surface receptor proteins in peripheral blood NK cells. In peripheral NK cells, a dramatic decrease in the expression of the activating receptor NKG2D was observed between COVID negative and positive groups as evidenced by a 35% percent decrease in expression for all three COVID-19 positive severity groups (p=0.03 negative vs. asymptomatic, p=0.0018 negative vs. MM, p=0.0088 negative vs. SC) (**Figure 2A**). Additionally, decreased expression of the inhibitory receptor KIR3DL1/DS1 was observed between the COVID-19 negative and COVID-19 positive groups. For example, the MM COVID-19 patients had a 51% decrease in expression as compared to COVID-19 negative patients (p=0.0025) (**Figure 2B**).

The remaining NK cell markers tested (CD16, NKG2C, NKp46, NKG2A, KIR2DL1, and KIR2DL2) did not show any significant differences when comparing COVID positive and COVID negative subjects or across COVID disease severity groups. A trend was observed for decreased NKG2C in COVID-19 positive patients, however, these results were not statistically significant.

### Dimensionality reduction analysis of NK cells separates COVID-19 positive from COVID-19 negative patients

**Figure 3:**
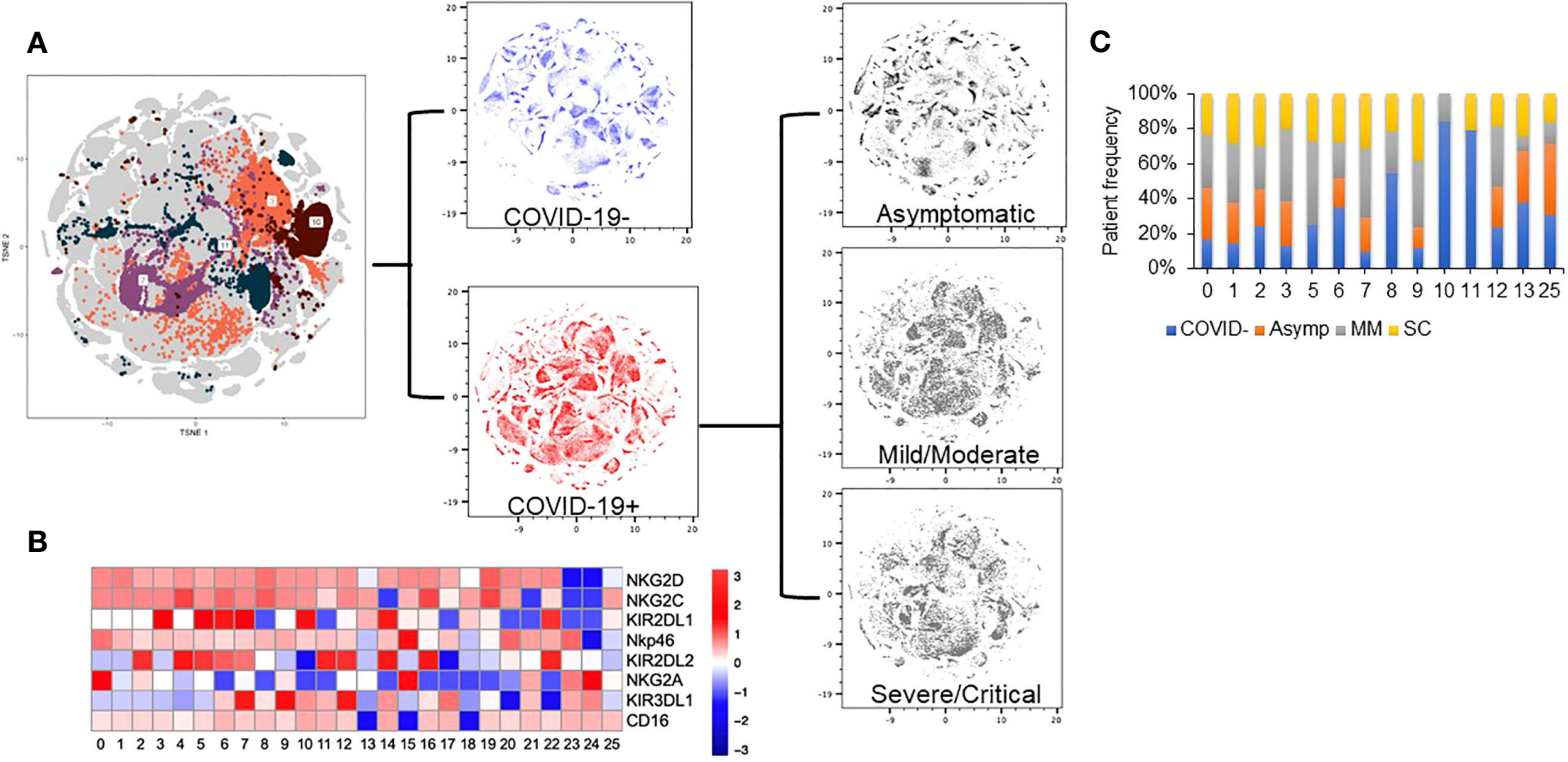
Dimension reduction analysis of multi-color flow panel in NK cells identifies changes in t-SNE distribution with disease severity. **(A)** Clustering analysis identifies 53 unique clusters based on the 10-color flow panel. Density plot of peripheral blood NK cells from 25 healthy donors (blue). Density plot of peripheral blood NK cells from 74 patients with COVID-19 (red). Density plot of peripheral blood NK cells from asymptomatic patients (top grey), mild/moderate patients (middle grey), and severe/critical patients (bottom grey). **(B)** Truncated heat map of protein expression of surface receptors on peripheral blood NK cells from patients with and without COVID-19 based on 5+ patient cuttoff per cluster. **(C)** Normalized patient frequency by disease severity per cluster. Clusters are shown based upon a cutoff of at least 5 patients expressing at least 10% of sample per cluster.

As NK cells are highly heterogenous, we next performed an unsupervised clustering analysis using the NK receptor expression data to identify specific clusters of NK cells differing amongst the cohorts. This analysis led to the identification of 53 unique clusters (**Figure 3A**). Topographic differences were visible in comparing the clusters identified based upon disease severity (**Figure 3A**). These distinct clusters allowed us to identify and describe NK cell subpopulations and identify the phenotype that defines these clusters (**Figure 3B**). We next assessed for clusters that were enriched in either COVID positive or COVID negative patients. We calculated the percent of cells from each patient, determined the patient frequency per cluster, and normalized to the total number of patients per severity group. Using a sample cutoff of at least 10% cell count per patient, and a sample size of over 5 patients per cluster, we found that Clusters 3 and 7 were highly enriched in COVID-19 positive patient NK cells (p value= 0.045 and 0.018 respectively), whereas clusters 10 and 11 were enriched in healthy control NK cells (**Figure 3C**) (p values= 0.003 and 0.018 respectively). For example, Cluster 3 was composed of 16% COVID-19 negative and 85% COVID-19 positive patients and Cluster 7 was 18% COVID-19 negative and 82% COVID-19 positive patients.

### NKG2A+ KIR2DL1+ and NKG2C- NK cells are associated with COVID-19

**Figure 4:**
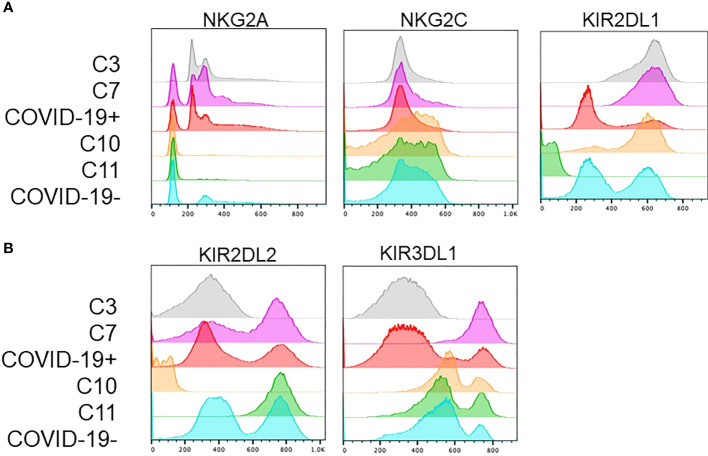
Clusters enriched in COVID-19 positive or COVID-19 negative patient NK cells exhibit distinct phenotypes. **(A)** COVID-19 positive enriched clusters (C3-grey and C7-pink) are found to be NKG2A+KIR2DL1+NKG2C-. COVID-19 negative enriched clusters (C10-orange and C11-green) are NKG2A-NKG2C+ with variable KIR2DL1 expression. **(B)** KIR2DL2 and KIR3DL1 expression was variable across the 4 clusters of interest.

Interestingly, the NK cells found within COVID-19 positive enriched clusters 3 and 7 elucidate a disease specific phenotype in circulating NK cells. Both clusters are characterized by high NKG2A and KIR2DL1 expression and low NKG2C expression (**Figure 4A**). COVID-19 negative enriched clusters (C10 and C11) are characterized by low NKG2A, high NKG2C and variable KIR2DL1 expression. KIR2DL2 and KIR3DL1 were variable across the 4 clusters of interest (**Figure 4B**). Given the known inhibitory roles of NKG2A and KIR2DL1, NK cells in clusters 3 and 7 may be less activated, and therefore less cytotoxic. This single cell analysis contrasts to the bulk cell analysis shown in **Figure 3** that is not able to elucidate these specific phenotypic differences among the patient cohorts. As the defining features of these clusters enriched in COVID-19 positive patients are high expression of the inhibitory molecules NKG2A and KIR2DL1, we next aimed to assess if blocking these molecules could enhance the activity of NK cells derived from COVID-19 patients against SARS-CoV-2 infected cells.

**Figure 5:**
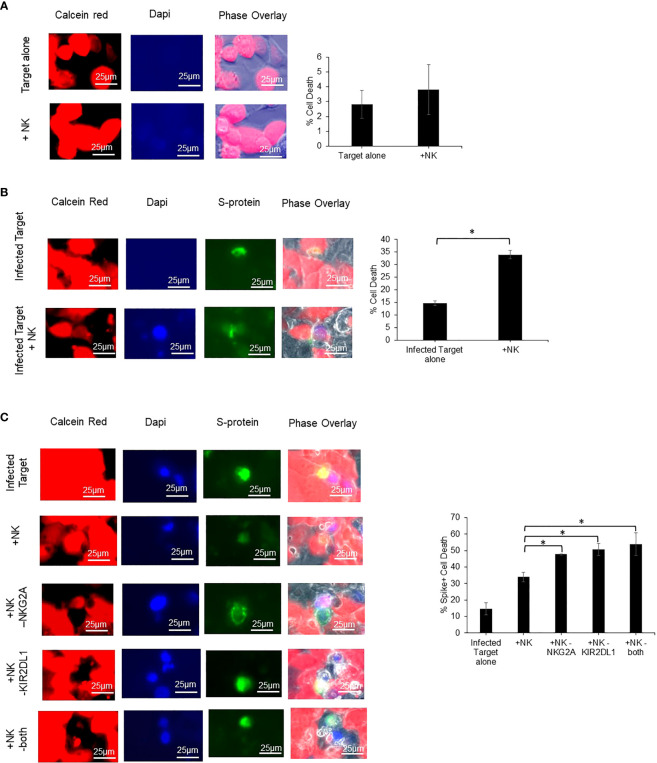
Blockade of NKG2A and KIR2DL1 enhances NK clearance of SARS-CoV-2 infected Caco2 cells. **(A)** Uninfected Caco-2 cell line does not undergo cell death after 2-hour co-culture with primary human NK cells. Red= Calcein (live cells) Blue= dapi (dead cells) (n=3). **(B)** Infected Caco-2 cells show greater loss of viability signal after co-culture with NK cells from COVID-19 positive donor derived NK cells. Green=S-protein (infected cells) (n=3). **(C)** Single and combination blockade of inhibitory receptors NKG2A and KIR2DL1 enhances cytotoxicity of COVID-19 positive donor derived NK cells against infected Caco-2 cells (n=3). *P<0.05.

Caco-2 cells were utilized as a target cell for assessing NK cell cytotoxicity as it is a human cell line that expresses ACE2, HLA-E, and HLA-C2. ACE2 is known to be required for SARS-CoV-2 infection, while HLA-E and HLA-C2 are known to be ligands for the receptors NKG2A and KIR2DL1 respectively (18–20). Prior to performing NK cell cytotoxicity assays, we confirmed the expression of NKG2A and KIR2DL1 on the NK cells derived from COVID-19 positive patients utilized for these assays by flow cytometry (**Supplemental Figure 1**). As NK cells are thought to lead to enhanced lysis of virally infected cells, we next confirmed that the NK cells lead to enhanced killing of SARS-CoV-2 infected Caco-2 cells as compared to non-infected cells. Non-infected Caco-2 cells had very low killing (~4%) by healthy donor derived NK cells that was similar to the amount of cell death observed in uninfected Caco-2 control cultures (**Figure 5A**). We next infected Caco-2 cells with SARS-Cov-2 and performed analysis specifically in infected cells as determined by S-protein expression. Using an MOI of 30, we achieved a 12% infection efficiency. We observed a 20% increase in the cytotoxicity of the SARS-Cov-2 infected Caco-2 cells that were co-cultured with COVID-19 positive patient derived NK cells as compared to infected Caco-2 cells alone (p=0.014) (**Figure 5B**). After establishing that NK cells exhibit enhanced cytotoxicity against SARS-CoV-2 infected Caco-2 cells as opposed to non-infected cells, we assessed if blockade of NKG2A, KIR2DL1 or a combination can enhance the NK cell-mediated cytotoxicity using blocking antibodies (n=3 COVID-19 positive donors). As seen in **Figure 5C**, there was a significant enhancement of COVID-19 positive patient derived NK cell killing of SARS-CoV-2 infected Caco-2 cells with the addition of either NKG2A or KIR2DL1 blockade (p<0.05). In contrast, the percent killing of the Spike protein negative cells (SARS-CoV-2 negative) in the same samples was unchanged (**Supplemental Figure 2**). Finally, we also assessed the ability of NKG2A and KIR2DL1 blockade to enhance the cytotoxic activity of NK cells from patients who were COVID-19 negative against spike expressing infected CACO-2 cells (**Supplemental Figure 3**). In contrast to testing NK cells from patients with COVID-19, these NK cells did not exhibit a statistically significant increase in killing with NKG2A blockade but did exhibit increased killing with KIR2DL1 blockade.

## Discussion

NK cells are amongst the first line of defense against viral pathogens. Their unique ability to recognize virally infected or malignantly transformed cells independent of antigen presentation enable them to lyse target cells rapidly and potently. To better understand the role of NK cells in COVID-19 infections as well as reveal potential new strategies to develop NK therapeutics, it is critical to better understand NK cells in the context of COVID-19 disease. Using a multi-colored flow cytometry panel and unbiased dimension reduction analysis, we characterized the NK cell phenotype after SARS-CoV-2 infection in patients with various COVID-19 severities including asymptomatic, mild/moderate, and severe/critical as well as healthy controls. Consistent with previous reports, we identified low NK, T, and total lymphocyte cell counts in patients with COVID-19, particularly patients with more severe disease. By traditional bulk analysis, we found a decrease in expression of the activating receptor NKG2D with increased disease severity. Additionally, there was trend towards a reduced expression of the activating receptor NKG2C in COVID-19 positive patients as compared to the negative control. Finally, the inhibitory KIR3DL1 protein was found at higher expression levels in the COVID-19 negative controls as compared to the disease cohort.

Unsupervised high-dimensional analysis identified unique clusters of NK cells linked to patients with COVID-19 and healthy controls. By evaluating these clusters, we identified an NK cell surface receptor expression signature that was predominantly observed in patients with or without COVID-19. Clustering analysis identified unique COVID-19 positive clusters to be NKG2A+KIR2DL1+NKG2C-. Given the expression of inhibitory NKG2A and KIR2DL1, in combination with the decrease in activating NKG2C, this phenotype is specific to a less cytotoxic NK cell. This finding is particularly interesting because it suggests an inhibition of NK cell activity as a mechanism for COVID-19 disease development. It has been previously reported that NKG2A inhibition, can lead to enhanced NK cell degranulation and NK cell cytotoxicity when NK cells are co-cultured with cells modified to express the SARS-CoV-2 spike protein (12, 13).

Our results are in line with the current body of literature, wherein we observed lymphopenia with more severe COVID-19, an increase in killing with NKG2A blockade, as well as a novel functional finding probing the efficacy of KIR2DL1 blockade with or without NKG2A blockade combination *in vitro* in improving COVID-19 derived NK cell antiviral cytotoxicity (9, 10, 12, 13). These results suggest that upregulated inhibitory receptors on NK cells may lead to NK cell inactivity, allowing virus to propagate and multiply. We demonstrated the possible therapeutic use of anti-NKG2A and/or anti-KIR2DL1 to restore NK function in COVID-19 patients.

In sum, these results provide a landscape of NK cell phenotype in COVID-19 patients. These results shed light onto the innate immune response in the context of SARS-CoV-2 infection. Currently NK cells as a therapeutic option are being explored in phase I/II clinical trials and the utility of combining anti-NKG2A and/or anti-KIR2DL1 in this context is also worthy of further investigation.

## Methods

### Surface receptor flow cytometry

Samples were acquired, processed, and analyzed on the same day to minimize inter-experiment variability. All samples were assigned a disease severity based on the worst symptoms present at the time of blood draw according to National Institutes of Health COVID-19 Treatment Guidelines Panel, available at https://www.covid19treatmentguidelines.nih.gov/. Discarded blood samples were obtained from patients from August 2020-April 2021 at the Louis Stokes Cleveland Medical Center, Cleveland, Ohio, USA. Peripheral blood samples from patients were directly stained with the following antibodies CD159a (FITC, Miltenyi), CD158e (APC, Miltenyi), CD314 (PE-VIO 615, Miltenyi), CD335 (PE, Beckman Coulter), CD3 (Krome-Orange, Beckman Coulter), CD56 (APC-Alexa Flour 700, Beckman Coulter), CD94 (Pacific blue, Beckman Coulter), CD19 (APC-Alexa Flour 750, Beckman Coulter), CD158b1/b2 (PC7, Beckman Coulter) and CD158a,h (PC5.5, Beckman Coulter). Samples were run on a Beckman Coulter Navios EX Flow Cytometer. Flow data analysis was performed using FlowJo 10.

### Unbiased cluster analysis

NK cells were identified by gating on the lymphocyte population *via* SSC vs. FSC, then by CD3-CD56+ expression prior to being imported into R v4.1.0. Mean fluorescence intensity (MFI) values were z-scaled. Using these z-scaled features as input, a t-SNE dimension reduction was calculated (21). A shared nearest-neighbors graph was calculated using the FindNeighbors function in Seurat v4 (22) and 53 low-resolution louvain clusters were found using the FindClusters function with a resolution of 0.06.

### Isolation and culture of NK cells

Naïve NK cells from peripheral blood patient samples were isolated using the StraightFrom Whole Blood CD56 MicroBeads (Miltenyi Biotec). Cells were cultured in 10% cosmic serum, 1% Penicillin/streptomycin, 0.1% amphotericin RPMI 1640 media with 50 units/ml IL2 for 24 hours until the assay was performed. Cells were incubated in an incubator at 37°C and 5% CO2.

### Cell culture

Caco-2BBE cells were cultured in RPMI 1640-L-glutamine supplemented with 10% cosmic serum, 1% penicillin/streptomycin, 0.1% amphotericin B, and 0.1% ciprofloxacin. Cells were incubated in an incubator at 37°C and 5% CO2.

### Viral culture

SARS-related Coronavirus 2 isolate (purchased from BEI) were filtered through 0.2 uM syringe filter and used to transduce Caco-2BBE cells in monolayer T-flasks.100% confluent Caco2-BBE cells were infected with an MOI of 30 for 1hr at 37°C and 5% CO2 in serum free media, after which fresh media was supplemented on top. Infected cells were used 24 hours after infection for assays.

### Quantification of viral titer

Filtered virus from BEI (NR-52282) was propagated in the same cell line. Cultured virus was heat-inactivated at 60°C for 30 minutes and titrated using qPCR. The standard curve was calculated using a known heat inactivated viral stock NR-52286 (BEI). Quantification of the virus was performed in a similar fashion as previously described (23).

### Cytotoxicity assay

NK cells were treated with 10μg/mL of NKG2A blocking antibody (Miltenyi Biotec #130-122-329) for 1hr and/or 10µg/mL KIR2DL1 blocking antibody (BD #556061) for 20 minutes in an incubator at 37°C and 5% CO2. NK cells were co-cultured with target cells in duplicate at a 1:1 ratio NK to target cell ratio. After a 2hr co-culture, the supernatant was removed, and the remaining attached cells were stained with 1µg/mL Calcein red-orange (Invitrogen) according to the manufacturers protocol. To detect the SARS-Cov-2 infected cells, the cells were blocked for 30 minutes at room temperature with 3%FBS/PBS and stained with S-Protein antibody (R&D systems Clone# 1035423) and a secondary goat anti-mouse IgG (H&L) AF488 (Invitrogen #A-11017). The cells were then fixed with 4% formaldehyde for 20 minutes at room temperature. Fixed cells were stained with 300nM Dapi for 5 minutes at room temperature and washed before imaging. Images were captured using a 40x objective in 4 fields per well using the Keyence bz-x800 (Keyence). We captured 1 picture per dye, as well as an overlay photo of all the fluorescent signal on top of the phase image. Captured fluorescent images were analyzed using the Keyence bz-x800 analyzer hybrid cell counter function. We first quantified bright DAPI positive cells, and then the Spike positive cells. Additionally, we counted the number of cells that were both DAPI and Spike positive, indicative of a dead/dying SARS-Cov-2 infected target cells. Using the calculated cell counts, we determined the %Spike positive cell death = #DAPI and Spike positive cells/# total Spike positive cells * 100.

### Statistics

Statistics were calculated using Graphpad Prism V9 or R v4.1.0 using the aov function with *post-hoc* Tukey’s test with Honest Significant Difference *via* TukeyHSD in the R package stats. The ANOVA test followed by the *post-hoc* Tukey’s test was applied to show the significant difference in the results found. *P<0.05, **P<0.01, ***P<0.001, ****P<0.0001

## Data availability statement

The raw data supporting the conclusions of this article will be made available by the authors, without undue reservation.

## Ethics statement

The studies involving human participants were reviewed and approved by Louis Stokes Cleveland VA IRB. Written informed consent for participation was not required for this study in accordance with the national legislation and the institutional requirements.

## Author contributions

GL, SV, and DW wrote the manuscript. DW and HS guided experimental design and data analysis. GL, JBo, and JBu performed the experiments. GL, MG, ZJ, LS, and RS analyzed data. MN and QA provided essential resources and support. All authors contributed to the article and approved the submitted version.
