# Supplementary figures and images for: NK cells from COVID-19 positive patients exhibit enhanced cytotoxic activity upon NKG2A and KIR2DL1 blockade
Source: Front Immunol. 2023 Jul 7;14:1022890. doi: 10.3389/fimmu.2023.1022890 (PMC10360118; doi:10.3389/fimmu.2023.1022890)

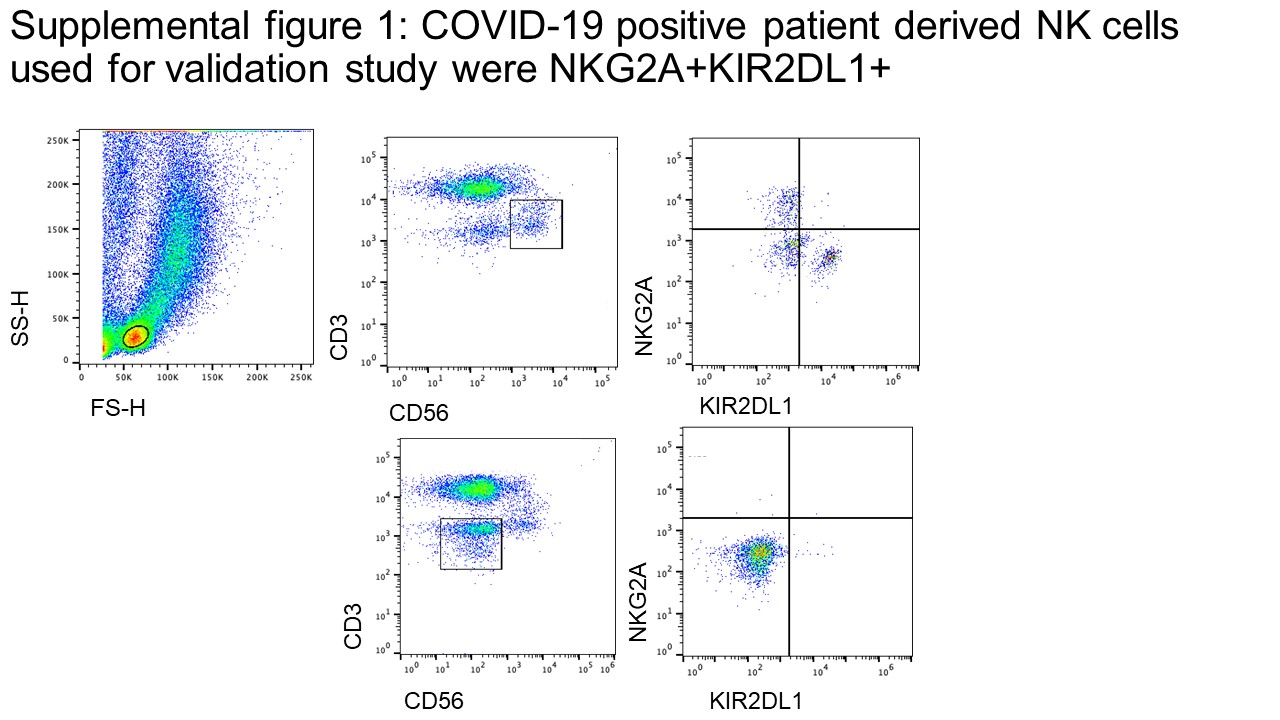

Supplement: Supplementary file 1 [file Image_1.jpeg]

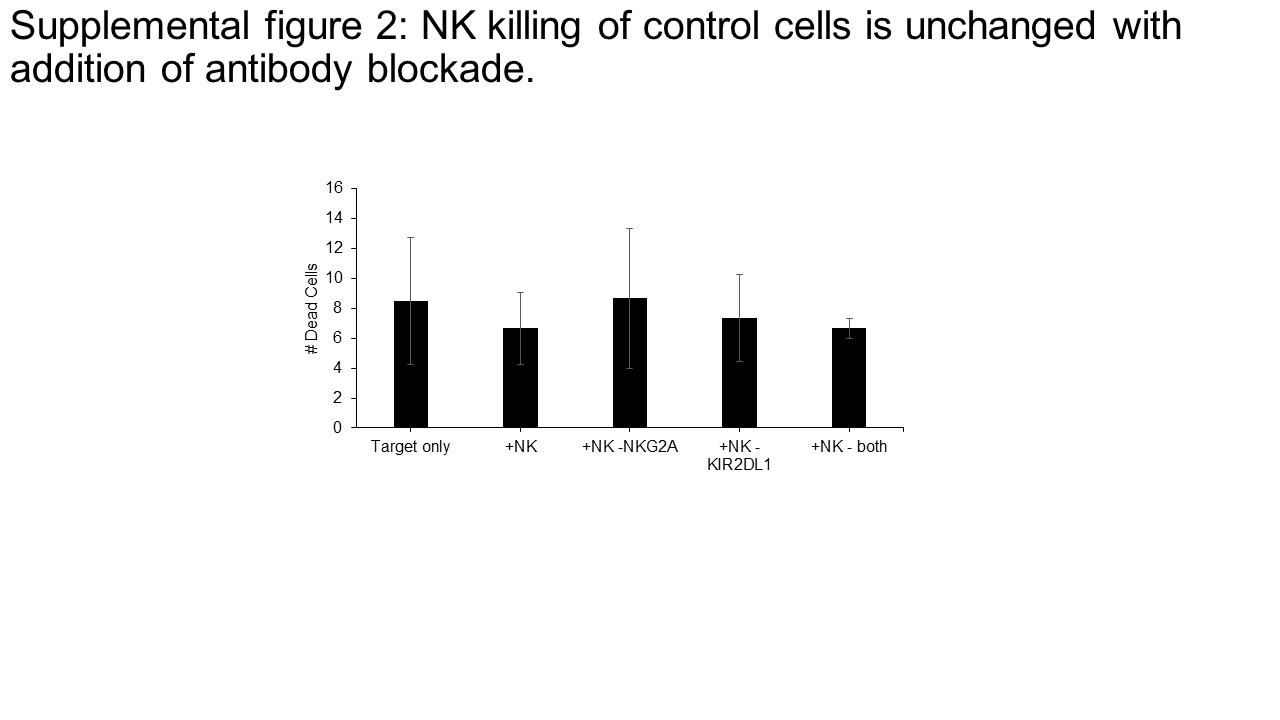

Supplement: Supplementary file 2 [file Image_2.jpeg]

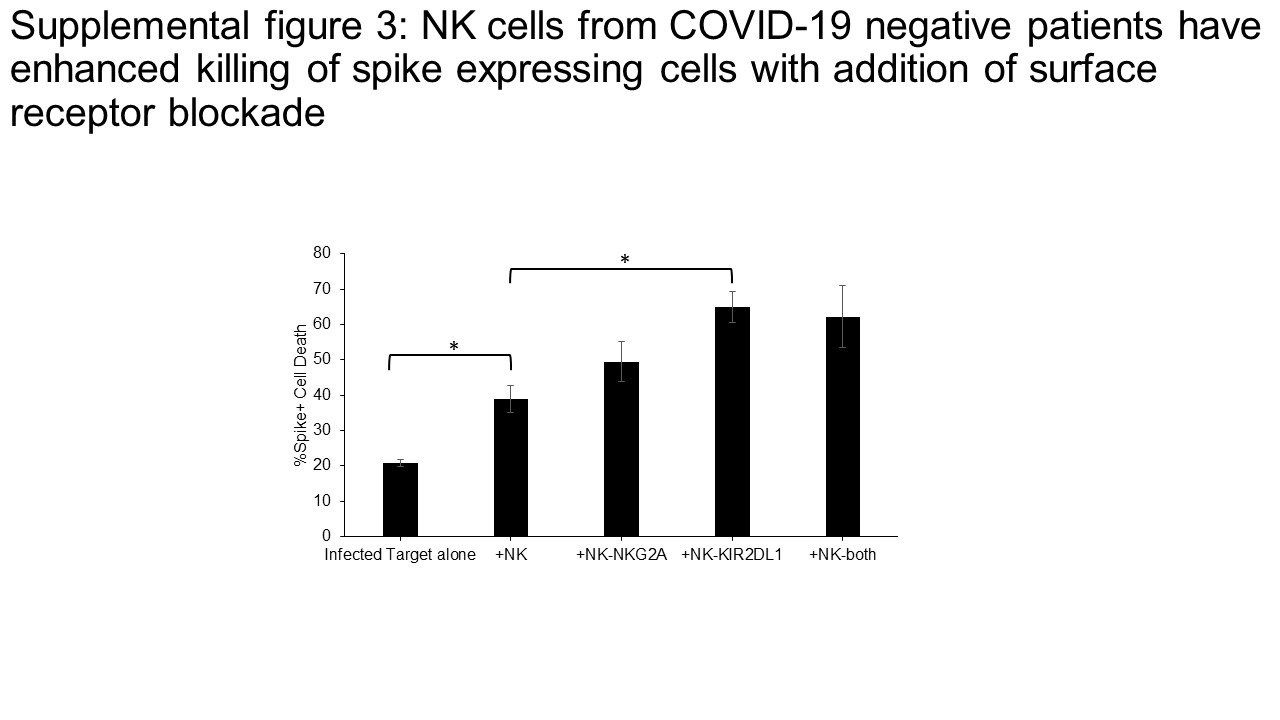

Supplement: Supplementary file 3 [file Image_3.jpeg]
